# Amiodarone and acupuncture for cardiac arrhythmia: Study protocol for a systematic review

**DOI:** 10.1097/MD.0000000000014544

**Published:** 2019-02-15

**Authors:** Xiang-Dong Meng, Wei-Qin Gao, Ze Sun

**Affiliations:** First Ward of Cardiology Department, First Affiliated Hospital of Jiamusi University, Jiamusi, China.

**Keywords:** acupuncture, amiodarone, cardiac arrhythmia, efficacy, randomized controlled trial, safety

## Abstract

**Background:**

Amiodarone and acupuncture (AA) are commonly used to treat cardiac arrhythmia (CA). The objective of this systematic review is to assess the efficacy and safety of AA for patients with CA.

**Methods:**

Randomized controlled trials (RCTs) of AA for CC will be searched from 9 databases including PubMed, EMBASE, Cochrane Library, Web of Science, Scopus, Chinese Biomedical Literature Database, China National Knowledge Infrastructure, VIP Information, and Wanfang Data from inception to February 1, 2019 without any limitations. Two reviewers will independently screen the relevant papers, extract data, and evaluate the risk of bias for each included study. RevMan 5.3 software will be used for meta-analysis. The primary outcome includes arrhythmic episodes (including time and frequency domain parameters). The secondary outcomes consist of health-related quality of life, oxygen saturation, and safety.

**Results:**

The protocol of this proposed study will provide evidence to judge whether AA is an effective treatment for patients with CA.

**Conclusion:**

The findings of this proposed study will summarize the up-to-date evidence of AA for CA.

**PROSPERO registration number:**

PROSPERO CRD42019120962.

## 1 Introduction

Cardiac arrhythmia (CA) is a very common disorder for patients with cardiovascular disease, which is often characterized as any change from the normal sequence of electrical impulses.^[1–3]^ Many factors are account for this disorder, such as smoking, high blood pressure, diabetes, high cholesterol, obesity, a high-fat diet, drug abuse, stress, family history, and so on.^[4–10]^ It has been reported that about 2% to 3% of the population in Europe and North America experienced such condition in 2014.^[11]^ Further studies found that approximately 50% deaths resulted from cardiovascular disease and 15% of all these deaths occurred because of the sudden cardiac death.^[12]^ Moreover, of these sudden cardiac deaths, about 80% of them are caused by CA.^[12]^ Thus, it is very important and very necessary to prevent and treat CA.

A variety of clinical studies have reported that the amiodarone, acupuncture, and combination of amiodarone and acupuncture (AA) are used to treat CA effectively.^[13–26]^ However, no systematic review and meta-analysis have been conducted to assess the efficacy and safety of AA for the treatment of CA. Thus, in this systematic review, we will explore the efficacy and safety of AA for the treatment of patients with CA.

## 2 Methods and materials

### 2.1 Inclusion and exclusion criteria

#### 2.1.1 Study types

This proposed systematic review will include randomized controlled trials (RCTs) that have evaluated all types of AA for CA. However, any other studies, such as non-clinical trials, case studies, non-RCTs, and quasi-RCTs will not be included.

#### 2.1.2 Participants

All participants of clinically diagnosed with CA will be included without restrictions of race, gender, and age.

#### 2.1.3 Interventions

The patients in the experimental group must receive any forms of AA, and will not combine with other treatments. The patients in the control group can receive any types of treatments, but not any forms of AA.

#### 2.1.4 Outcomes

The primary outcome includes arrhythmic episodes (including time and frequency domain parameters). The secondary outcomes are health-related quality of life, oxygen saturation, and safety.

### 2.2 Literature search strategy

Nine databases will be searched from inception to February 1, 2019 without any restrictions. These databases include PubMed, EMBASE, Cochrane Library, Web of Science, Scopus, Chinese Biomedical Literature Database, China National Knowledge Infrastructure, VIP Information, and Wanfang Database. All relevant RCTs of AA for CC will be fully considered in this study. Moreover, the websites of clinical registration and reference lists of included trials will also be searched to avoid missing any potential trials. The details of the search strategy are shown in Table 1, and a similar strategy will be performed to other databases.

**Table 1:**
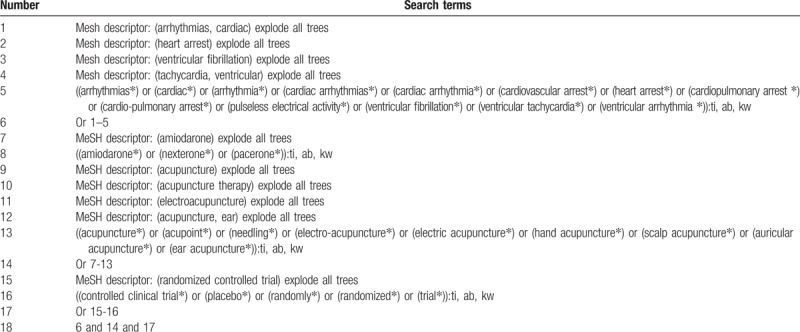
Search strategy applied in Cochrane Library database.

### 2.3 Study selection

**Figure 1:**
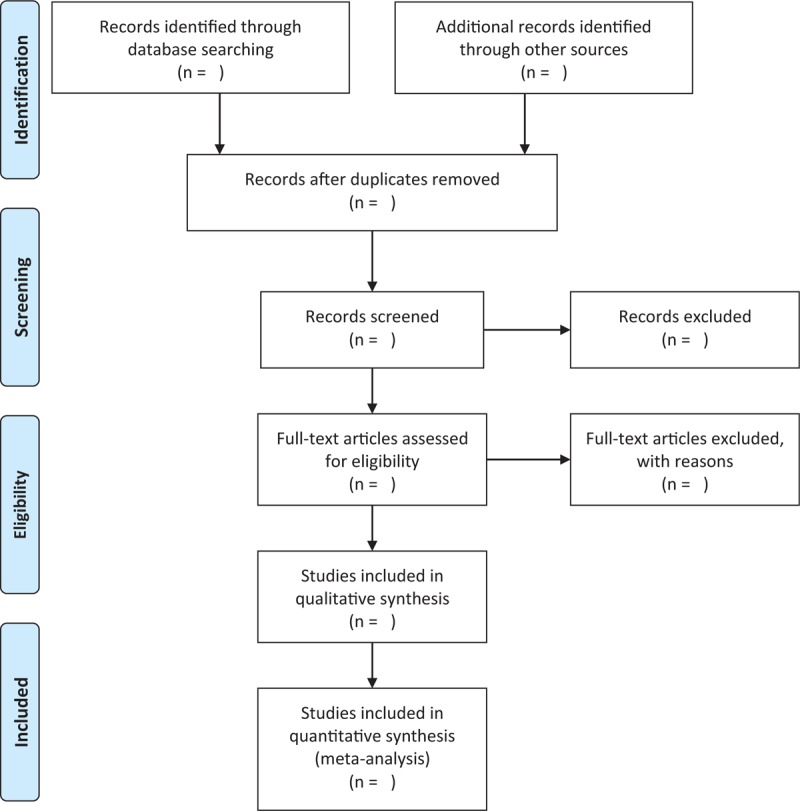
Flowchart of study selection.

Two independent authors will select studies by scanning titles and abstracts initially, and then full-texts will be further read if it is necessary. Any potential studies will be fully considered. The whole procedure of study selection is shown in Figure 1. The disagreements regarding the study selection will be solved through discussion with a third author.

### 2.4 Data extraction and management

All data will be extracted by 2 independent authors following predefined standard form of data extraction. Any divergences will be resolved by consulting a third author. The form consists of the following information.

(1) General information: title, first author, country, year of publication;
(2) Basic characteristics: sample size, gender, age, eligibility criteria of diagnosis, inclusion and exclusion, and other specific details;
(3) Methods: study design, sample size, sequence generation, allocation and concealment, blinding, and any other bias;
(4) Treatment details: details of experimental and controls, dose, duration, and frequency of interventions;
(5) Outcomes: All primary and secondary outcomes, and any other reporting information.

### 2.5 Dealing with missing data

We will contact primary authors if any insufficient or missing data will happen duration the data extraction period. If those missing data are not available, we will only analyze the available data, and will discuss their potential effects.

### 2.6 Methodological quality assessment

Cochrane Risk of Bias Tool will be used to assess the methodological quality by 2 independent authors. This tool includes 7 fields, and each aspect will be judged as high risk of bias, or unclear risk of bias, or low risk of bias. Any divisions will be resolved by consulting a third author for the methodological quality assessment.

### 2.7 Statistical analysis

ReMan 5.3 software will be utilized for statistical analysis. All the extracted data will be categorized into continuous variables, presented as the mean difference or standardized mean difference with 95% confidence intervals; or dichotomous variables, as showed with risk ratio and 95% confidence intervals.

Heterogeneity among trials is determined by using *I*^*2*^ test. A fixed-effects model will be used to pool the data for assessing the effects of AA for CA if *I*^2^ ≤50%. Otherwise, a random-effects model will be utilized to pool the data. Where significant heterogeneity is detected, subgroup analysis will be carried out based on the different study characteristics, experimental therapies, control treatments, and outcome measurements. A narrative summary will be elaborated if substantial heterogeneity is still detected after the subgroup analysis, and data will not be pooled. In addition, sensitivity analysis will be performed to check the robustness of pooled data by removing low-quality trials. Moreover, funnel plot,^[27]^ and Egg regression^[28]^ will be conducted to detect the reporting biases if more than 10 trials are included.

## 3 Discussion

Amiodarone has been used to treat CA for a long time. However, it still has limited efficacy and also accompanies lots of adverse events for long term treatment. Thus, adjunctive therapy with more exciting efficacy and fewer adverse events to amiodarone is urgently needed for the treatment of CA. Fortunately; many CA patients in China are seeking traditional Chinese medicine for their alternative treatments, and have achieved promising outcome results. For example, acupuncture, as one of the most important parts of traditional Chinese medicine has long been used for the treatment of CA condition in China. However, no systematic review has investigated the efficacy and safety of AA for CA. Thus, in this systematic review, we first assessed the efficacy and safety of AA for the treatment of CA. The results of this study will provide the first rigorous summary evidence of AA for CA across all published RCTs.

The data pooled will provide a better understanding of the efficacy and safety of AA for patients with CA. Its findings will inform our understanding of the value of AA in treating CA outcomes. Additionally, it may also provide helpful evidence for clinical practice and future studies.

## Author contributions

**Conceptualization:** Xiang-Dong Meng, Wei-Qin Gao, Ze Sun.

**Data curation:** Xiang-Dong Meng, Wei-Qin Gao, Ze Sun.

**Formal analysis:** Xiang-Dong Meng, Ze Sun.

**Funding acquisition:** Xiang-Dong Meng, Wei-Qin Gao.

**Investigation:** Wei-Qin Gao.

**Methodology:** Xiang-Dong Meng, Ze Sun.

**Project administration:** Wei-Qin Gao.

**Resources:** Xiang-Dong Meng, Wei-Qin Gao, Ze Sun.

**Software:** Xiang-Dong Meng, Ze Sun.

**Supervision:** Wei-Qin Gao.

**Validation:** Xiang-Dong Meng, Wei-Qin Gao, Ze Sun.

**Visualization:** Xiang-Dong Meng, Wei-Qin Gao, Ze Sun.

**Writing** – **original draft:** Xiang-dong Meng, Wei-Qin Gao, Ze Sun.

**Writing** – **review and editing:** Xiang-Dong Meng, Wei-Qin Gao, Ze Sun.
